# Endoscopic Sinus Surgery Significantly Reduces Eustachian Tube Dysfunction Symptoms in Patients with Chronic Rhinosinusitis: A Systematic Review and Meta-Analysis

**DOI:** 10.3390/biomedicines12112484

**Published:** 2024-10-29

**Authors:** Kai-Shan Yang, Wei-Chih Chen, Ching-Nung Wu, Yin-Shen Wee, Ching-Shuen Wang, Cheng-Chih Wu, Sheng-Dean Luo

**Affiliations:** 1 Department of Otolaryngology, Kaohsiung Chang Gung Memorial Hospital, Chang Gung University College of Medicine, Kaohsiung 833, Taiwan; ks307@cgmh.org.tw (K.-S.Y.); jarva@cgmh.org.tw (W.-C.C.); taytay@cgmh.org.tw (C.-N.W.); 2 Department of Public Health, College of Medicine, National Cheng Kung University, Tainan 701, Taiwan; 3 Department of Pathology, University of Utah, Salt Lake City, UT 84112, USA; yinshenwee@tmu.edu.tw; 4 School of Dentistry, College of Oral Medicine, Taipei Medical University, Taipei 110, Taiwan; chingshuenwang@tmu.edu.tw; 5 Department of Pharmacy, Kaohsiung Chang Gung Memorial Hospital, Kaohsiung 833, Taiwan; 6 School of Pharmacy, Kaohsiung Medical University, Kaohsiung 807, Taiwan; 7 School of Traditional Chinese Medicine, Chang Gung University College of Medicine, Taoyuan 33302, Taiwan; 8 Graduate Institute of Clinical Medical Sciences, College of Medicine, Chang Gung University, Taoyuan 33302, Taiwan

**Keywords:** Eustachian tube dysfunction, chronic rhinosinusitis, endoscopic sinus surgery, systematic review, meta-analysis, ETDQ-7

## Abstract

(1) Background: Eustachian tube dysfunction (ETD) is a frequently observed clinical manifestation of chronic rhinosinusitis (CRS). This systematic review aimed to evaluate the function of the Eustachian tube following endoscopic sinus surgery (ESS) in adult CRS patients with confirmed preoperative ETD symptoms. (2) Methods: A systematic search of PubMed, Cochrane, Embase, and MEDLINE electronic databases was conducted. The review was performed following the PRISMA guidelines. Studies investigating concurrent ETD in CRS patients who underwent ESS were retrieved. The changes in ETD outcomes were measured by the 7-item Eustachian Tube Dysfunction Questionnaire (ETDQ-7). We employed a random-effects model to conduct the meta-analysis (3) Results: We included seven observational studies that involved a total of 436 CRS patients with concurrent ETD. The pooled results revealed a statistically significant reduction (Standardized mean difference = −1.24; 95% CI = −1.64 to −0.84) in ETDQ-7 scores among the CRS with ETD patient cohort at the 3-month postoperative follow-up. (4) Conclusions: ESS serves as an effective intervention for improving E-tube function in adult CRS patients with concurrent ETD. Future prospective randomized controlled trials that incorporate various outcome predictors should be conducted to explore potential clinical factors for greater ETD improvement and normalization after ESS.

## 1. Introduction

The Eustachian tube (E-tube) equalizes pressure across the tympanic membrane, supplies air to the middle ear for ventilation, and acts as a channel for mucociliary clearance and sound protection [1]. Paranasal sinuses and nasal cavities are situated in close proximity to the middle ear, which is connected to them by the E-tube. Therefore, considering its anatomic location, Eustachian tube dysfunction (ETD) is one of the more common otologic manifestations of chronic rhinosinusitis (CRS). The prevalence of otologic symptoms including aural fullness, hearing loss, otalgia, and tinnitus ranged from 15% to 42% in patients diagnosed with chronic rhinosinusitis [2]. The chronic inflammatory response of sinonasal mucosa can lead to the swelling of the E-tube orifice, which can result in the impairment of the pressure equalization and dilatory functions of the E-tube [3,4,5]. Patients who suffer from E-tube dysfunction can report a poorer quality of life due to persistent otologic symptoms. The latest version of the European Position Paper on Rhinosinusitis and Nasal Polyps (EPOS 2020) provides evidence-based recommendations for the management of rhinosinusitis, but gives limited attention to CRS-related ETD treatment [6]. Previous studies have investigated the effect of ETD symptoms following endoscopic sinus surgery in CRS patients [5,7]. However, the extent of ETD improvement across different countries has not been thoroughly explored. Therefore, this is the first systematic review to analyze the surgical impact on changes in ETD outcomes in CRS patients worldwide with confirmed preoperative ETD symptoms. The primary objectives of this study are to review the current literature on the prevalence of ETD in CRS and conduct a meta-analysis on the effect of endoscopic sinus surgery for CRS patients with concurrent ETD symptoms.

## 2. Materials and Methods

### 2.1. Search Strategy

The study was developed according to the latest version of Preferred Reporting Item for Systematic Reviews and Meta-Analyses (PRISMA) guidelines (Supplementary Table S1), [8] and was registered in INPLASY with the registration number (INPLASY202480056). Articles were identified through a comprehensive search of PubMed, Cochrane, Embase, and MEDLINE electronic databases. The search strategies used a combination of medical subject headings (MeSH) and keywords including (‘CRS’ OR ‘chronic rhinosinusitis’ OR ‘chronic sinusitis’ OR ‘sinusitis’ OR ‘rhinosinusitis’) AND (‘ETD’ OR ‘Eustachian tube dysfunction’ OR ‘E tube dysfunction’ OR ‘Eustachian tube’ OR ‘Eustachian tube function’) AND (‘ESS’ OR Endoscopic sinus surgery’ OR ‘nasal surgery’ OR Functional endoscopic sinus surgery’ OR ‘FESS’). The results were limited to the English language only. The search was conducted from the earliest record in each database to the date of inspection (13 March 2024). The detailed search strategy is outlined in Supplementary Table S1.

### 2.2. Study Selection

Studies describing concurrent ETD in patients with CRS treated by endoscopic sinus surgery were included in the final analysis. The enrolled subjects were adult CRS patients (age > 18) who had an unsatisfactory response to medical therapy and concurrent ETD, which was primarily measured by the 7-item Eustachian Tube Dysfunction Questionnaire (ETDQ-7). ETDQ-7 has been established as a reliable objective measurement for adult patients with ETD. A total item score of ≥14.5 yielded 100% sensitivity and 100% specificity for ETD [9]. Exclusion criteria were studies without either mean preoperative or postoperative ETDQ7 scores with standard deviations, patients receiving concurrent interventions other than ESS, previous history of oncologic management of the head and neck region, pre-existing otologic disease, and positive response to medical therapy. Descriptive studies or review articles published in languages other than English were also excluded.

### 2.3. Data Collection

Abstracts were first screened by two reviewers (K.S. Yang and W.C Chen) for full-text review, then each article was independently assessed for eligibility. A third reviewer (C.N. Wu) was consulted in situations where consensus was not achieved. Primary outcome measures were mean preoperative and postoperative ETDQ7 scores. The ETDQ7 consists of 7 items, with each question being rated on a scale from 1 to 7. Total scores range from 7 to 49, and a total item score cut point of ≥14.5 or a mean item score cut point of ≥2.1 indicates the presence of Eustachian tube dysfunction [9].

### 2.4. Quality Assessment

The methodological quality and content validity of the recruited studies is assessed using the Newcastle–Ottawa Scale (NOS), which is designed to evaluate the risk of bias for nonrandomized studies, including case–control and cohort studies. The NOS contains eight items, which are categorized into three broad perspectives: the selection of the study groups; the comparability of the groups; and the ascertainment of either the exposure (case–control studies) or outcome (cohort studies) of interest [10]. A star system is implemented to allow semi-quantitative assessment of study quality. Two authors (K.S Yang and W.C Chen) used this instrument for study appraisal by awarding a maximum of one star for each item in the selection and outcome section, and a maximum of two in the comparability section if it was fulfilled. Scores on the NOS vary between 0 and 9 points. Studies with a total score of >6 are considered to have good methodological quality. Consensus was reached through discussion when there was a discrepancy between ratings.

### 2.5. Statistical Analysis

Meta-analysis of continuous measures, namely mean preoperative and postoperative ETDQ7 scores, was performed with Cochrane Review Manager (RevMan) version 5.4 (The Cochrane Collaboration 2020). Due to the variation between the enrolled studies such as target populations and mean outcome measures, a random-effects model was used to run the analysis. The proportion was weighted according to the number of subjects in each study. The effect size of the primary study outcome was quantified using the standardized mean difference and 95% confidence intervals (CIs), since not all the included studies employed the same scale to measure ETDQ-7 scores. The degree of statistical heterogeneity across studies was evaluated by I^2^ and Cochrane’s Q test. I^2^ statistics of 25%, 50%, and 75% were estimated as low, moderate, and high heterogeneity, respectively [11].

We performed the sensitivity analysis through the one-study removal method to determine whether withdrawing any particular trial could cause a statistically significant difference in summary effect size [12]. Funnel plots were generated and visually inspected for analysis. Potential publication bias could be observed based on asymmetry of the funnel plot. Egger’s regression was not tested as there were less than 10 studies involved. A *p*-value of less than 0.05 was considered statistically significant for all statistical tests.

## 3. Results

### 3.1. Study Selection

**Figure 1:**
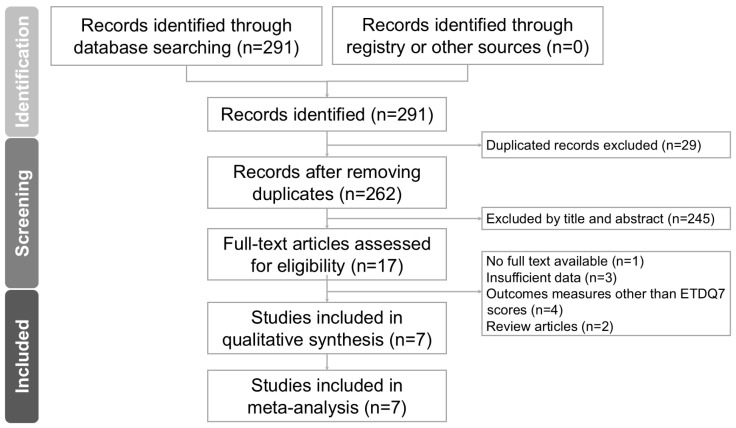
PRISMA flowchart for the current meta-analysis.

The literature search process is presented in the PRISMA flowchart (Figure 1). A total of 291 articles were identified through a systematic search of databases. Following the exclusion of 29 duplicate publications and 245 ineligible studies after title and abstract review, we retrieved 17 articles for full-text review. Seven observational studies ultimately met the criteria, and were included in the meta-analysis. Out of the 10 excluded articles in the final stage, one study had no full text available [2], while three studies had insufficient data, either missing postoperative mean ETDQ7 scores or standard deviations [4,13,14]. Four studies were excluded because ETD symptoms were measured with changes in SNOT-22 otologic subdomain scores or the type of tympanometry, thus disallowing useful comparisons to be drawn for the primary outcome [3,15,16]. The remaining two studies were systematic reviews [5,7]. Therefore, seven studies [17,18,19,20,21,22,23] were included in this systematic review and meta-analysis. All studies were observational in design, including five prospective cohort studies [17,19,20,21,22], one prospective case–control study [23], and one retrospective cohort study [18].

### 3.2. Baseline Characteristics

The summary of the included case series is presented in Table 1. A combined total of 436 patients from these seven studies had concurrent ETD. Four of the studies enrolled a total number of 533 CRS patients that underwent ESS from 2016 to 2019, whereas the remaining three [11,20,22] recruited 155 CRS patients with confirmed diagnoses of ETD admitted for ESS. The prevalence of ETD among CRS patients was 53% (281/533) based on the four studies that enrolled adult CRS patients regardless of ETD status initially. The postoperative evaluations were performed at a range of 2-month to 1-year period. We compared the ETDQ-7 scores at the 3-month interval after ESS as the basis for this analysis, except Higgins et al., which assessed the patients 2 months following operations. With regard to CRS metrics and comorbidities, 197 out of 606 (32.5%) patients had nasal polyposis in six studies. Concomitant allergic rhinitis was recorded in 126 out of 489 (25.7%) CRS patients from three studies. A total of 197 out of 524 (37.6%) patients received revised ESS as documented by four studies. The detailed summary of patient characteristics of the included studies is listed in Table 2.

### 3.3. Methodological Quality Assessment of the Included Studies

We presented the quality appraisal of studies using NOS in Table 3. All the included studies were considered to have good methodological quality (total scores > 6). No stars were awarded to two studies, Chang et al. and Hsieh et al., for the adequacy of the follow-up because they did not document whether any participants were lost to follow-up [18,22]. The funnel plot demonstrated all studies were within the funnel distribution except for two without symmetry, suggesting some publication bias based on visual inspection (Supplementary Figure S1).

**Table 1:** Summary of the retrieved trials investigating the effect of endoscopic sinus surgery on improving ETD in the enrolled participants.

First Author and Year	Country	Study Period	Study Design	Inclusion Criteria	Exclusion Criteria	Total CRS Patients (*n*)	CRS + ETD (*n*)	Definition of ETD	Pre-op ETDQ-7 Score	Post-op ETDQ-7 Score
Bowles 2019 [17]	United Kingdom	August 2016– November 2017	Prospective cohort study	Adult patients with refractory CRS despite a minimum of 6 months of medical therapy	Pre-existing otological disease, adequate response to medical therapy, patient preference for continuing medical therapy, contraindication to general anesthetic	57	39	ETDQ-7 ≥ 13.5	20.6 ± 10.34	11.4 ± 5.65
Chang 2020 [18]	United States	December 2016–December 2018	Retrospective cohort study	Adult patients diagnosed with CRS or RARS delineated by International Consensus Statement on Allergy and Rhinology	Known otologic comorbidities apart from ETD, prior otologic surgery, sinonasal diagnoses other than sinusitis	302	180	ETDQ-7 ≥ 14.5	25.5 ± 7.6	16.8 ± 8.5
Higgins, 2020 [23]	United States	November 2016–December 2017	Prospective case–control study	Adult CRS patients with persistent ETD without MEE despite a period of 6–8 weeks of maximum medical therapy	Ear surgery, adenoidectomy, Eustachian tube dilation, MEE, cholesteatoma, history of major ear surgery, severe atelectasis, congenital ear disorder, prior head and neck surgery	60	60	Mean ETDQ-7 ≥ 2.1	3.45 ± 1.061	2.164 ± 1.206
Wu, 2020 [21]	United States	September 2018–March 2019	Prospective cohort study	Adult patients with CRS after failed medical therapy according to International Consensus Statement on Allergy and Rhinology	Failure to complete preoperative ETDQ-7 and SNOT-22 questionnaires	82	39	ETDQ-7 ≥ 14.5	16.8 ± 8.2	12.7 ± 6.8
Chen, 2021 [20]	China	December 2019–December 2020	Prospective cohort study	Adult CRS patients with concurrent ETD and were refractory to conservative treatment for at least 12 weeks	Meniere’s disease, low-frequency sensorineural hearing loss, patulous Eustachian tube, chronic suppurative otitis media, acute upper respiratory infection and temporomandibular joint dysfunction	70	70	ETDQ-7 ≥ 14.5 + Tympanogram B/C ETDQ-7 ≥ 14.5 + ETS ≤ 5	20.13 ± 6.18	8.63 ± 3.62
Chen, 2022 [19]	Taiwan	December 2016–December 2017	Prospective cohort study	Adult CRS patients with unsatisfactory response to medical treatment for at least 2 months	History of radiotherapy of the head and neck region, ear surgery, pharyngeal surgery	92	23	ETDQ-7 ≥ 14.5	25.52 ± 7.7	10.6 ±8.31
Hsieh, 2024 [22]	Taiwan	July 2018–June 2022	Prospective cohort study ^1^	Adult CRS patients with at least ethmoid and maxillary sinuses involved and diagnosis of ETD for at least 3 months	Elderly individuals, pregnant women, history of head and neck cancer, previous ear surgery, septoplasty, turbinate reduction procedures, TMJ disorder, aspirin-exacerbated respiratory disease	25	25	ETDQ-7 ≥ 14.5 + Inflation–deflation: (*p*) ^2^ Grade II-IV endoscopic inflammation score	22.84 ± 7.39	16.24 ± 7.74
Total (*n*)	-	-	-	-	-	688	436		-	-

Abbreviations: CRS, chronic rhinosinusitis; Pre-op, preoperative; ETD, Eustachian tube dysfunction; ETDQ-7, 7-item Eustachian Tube Dysfunction Questionnaire; Post-op, postoperative; MEE: middle ear effusion; TMJ: Temporomandibular joint; ETS: Eustachian tube score. ^1^ Hsieh, 2024 was a prospective randomized controlled trial comparing the treatment effect of ESS alone with combined balloon Eustachian tuboplasty (BET) in patients with CRS and ETD. Only the ESS group was extracted from the study to be included in this meta-analysis for comparative purposes. Thus, we defined the study as prospective cohort study here. ^2^ (*p*) represents a poor result on the inflation–deflation test.

**Table 2:** Summary of the characteristics of recruited patients.

First Author and Year	Total Number of CRS Patients	Nasal Polyposis	Allergic Rhinitis	Asthma	Revised ESS
Bowles 2019 [17]	57	23	-	16	-
Chang 2020 [18]	302	60	63	114	136
Higgins, 2020 [23]	60	18	-	-	32
Wu, 2020 [21]	82	-	-	-	-
Chen, 2021 [20]	70	51	25	3	17
Chen, 2022 [19]	92	39	30	5	12
Hsieh, 2024 [22]	25	6	8	3	-
Total, *n* (%)	688	197 (32.5)	126 (25.7)	141 (25.8)	197 (37.6)

**Table 3:** Detailed quality assessment of the included studies using the Newcastle–Ottawa Scale (NOS) A. Cohort studies. B. Case–control study.

A. First Author	Selection				Comparability	Outcomes			Total
	Representativeness of Exposed Cohort	Selection of Nonexposed Cohort	Ascertainment of Exposure	Outcome not Present at the Start of the Study	Comparability Based on the Design or Analysis	Assessment of Outcomes	Length of Follow-Up	Adequacy of Follow-Up	
Bowles 2019 [17]	**✵**	**✵**	**✵**	**✵**	**✵**	**✵**	**✵**	**✵**	8
Chang 2020 [18]	**✵**	**✵**	**✵**	**✵**	**✵**	**✵**	**✵**	-	7
Wu 2020 [21]	**✵**	**✵**	**✵**	**✵**	**✵**	**✵**	**✵**	**✵**	8
Chen 2021 [20]	**✵**	**✵**	**✵**	**✵**	**✵**	**✵**	**✵**	**✵**	8
Chen 2022 [19]	**✵**	**✵**	**✵**	**✵**	**✵**	**✵**	**✵**	**✵**	8
Hsieh 2024 [22]	**✵**	**✵**	**✵**	**✵**	**✵**	**✵**	**✵**	-	7
B. First Author	Selection				Comparability	Exposure			Total
	Representativeness of cases	Selection of controls	Definition of controls	Adequate cases definition	Comparability based on the design or analysis	Ascertainment of exposure	Same method of ascertainment	Non-response rate	
Higgins 2020 [23]	**✵**	**✵**	**✵**	**✵**	**✵**	**✵**	**✵**	**✵**	8

Good quality: A total of 3 or 4 stars in the selection domain AND 1 or 2 stars in comparability domain AND 2 or 3 stars in the outcome/exposure domain. Fair quality: A total of 2 stars in the selection domain AND 1 or 2 stars in comparability domain AND 2 or 3 stars in the outcome/exposure domain. Poor quality: A total of 0 or 1 star in the selection domain OR 0 stars in comparability domain OR 0 or 1 stars in the outcome/exposure domain.

### 3.4. Primary Outcomes

**Figure 2:**
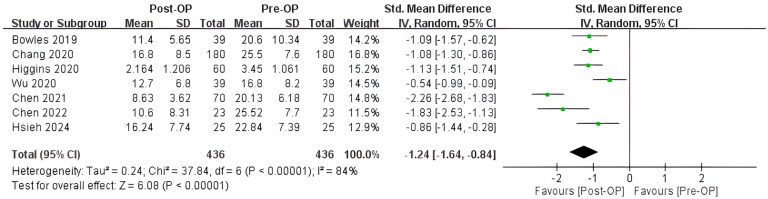
Forest plot showing the effect of endoscopic sinus surgery (ESS) on reducing ETDQ-7 scores in CRS patients. Surgical treatment of CRS was associated with relief of ETD symptoms. SD, standard deviation; Total, total number of subjects; Std, standard; CI, confidence interval; Op, operation; The green dot represents the point estimate of the study result. The horizontal line extending from each green dot represents the 95% confidence interval (CI) for the effect estimate. The black diamond symbol represents the overall effect estimate from all the studies included in the meta-analysis, with the width of the diamond reflects the 95% CI around the pooled effect estimate. Bowles 2019 [17]; Chang 2020 [18]; Higgins 2020 [23]; Wu 2020 [21]; Chen 2021 [20]; Chen 2022 [19]; Hsieh 2024 [22].

Following endoscopic sinus surgery, the ETDQ-7 score showed a statistically significant reduction (standardized mean difference = −1.24; 95% CI = −1.64 to −0.84, I^2^ = 84%) in the CRS with ETD patient cohort (Figure 2). High heterogeneity was observed nonetheless. A sensitivity analysis was conducted by employing the one-study-removal method [12]. No statistically significant change in the standard difference in mean was found when any one of the studies was excluded.

## 4. Discussion

According to previous studies, around 48.5% of CRS patients had clinically significant ETD, defined as an ETDQ-7 score of 14.5 or higher [4]. The preoperative prevalence rates of ETD in our enrolled studies varied from 25% to 68% [17,18,19,21]. This study corroborated the finding of ETD being a common comorbidity of CRS. Inflammatory changes and mucosal edema characterized in CRS may extend to the nasopharyngeal region, which could lead to swelling and obstruction of the E-tube orifice [24]. Impairment in E-tube dilatory function and increased nasal airway resistance give rise to negative middle ear pressure. The resulting pressure gradient contributes to ETD, which is associated with otologic manifestations, including aural fullness, cracking/popping, otalgia, and otitis media effusion [2,25,26]. Treating underlying sinonasal diseases may thereby alleviate ETD symptoms.

In this meta-analysis, ESS was shown to statistically reduce ETD symptoms in CRS patients, and statistical significance was maintained in the sensitivity analysis. A retrospective case series by Adams et al. found a mean reduction of 5.1 ± 8.4 points in the ETDQ-7 score among CRS participants with concurrent ETD after receiving ≥4 weeks of medical treatment [27]. Additionally, the pilot study by Stoikes et al. reported a significant postoperative treatment effect on all otologic symptoms associated with CRS [2]. Recent studies have sought to evaluate the surgical approach for CRS patients with concurrent ETD. Sinus epithelization is typically completed at 3–6 months following operation, when nasal mucosa has returned to its normal state [28]. Hence, the recruited studies were assessed and compared at a baseline of 3 months postoperatively. Based on this meta-analysis, endoscopic sinus surgery has proven to be effective in improving Eustachian tube function in CRS patients with ETD, as evidenced by a significant reduction in postoperative ETDQ-7 scores.

Although there was one prior meta-analysis that studied the impact of ESS on ETD in CRS patients, this is the first systematic review to date to recruit the largest number of case series from Asia, Europe, and the United States to demonstrate this promising effect. This suggests the applicability of our findings to both Eastern and Western populations, which may display differences in the inflammatory phenotypes and endotypes of CRS [29]. Compared to the Western population, a lower prevalence of CRS comorbid with eosinophilic disease was found in the East Asian population. This might reflect a lower inflammatory burden in the Eastern population [19]. Additionally, this is the first analysis to discuss the response of ETD improvement in CRS patients following ESS based on clinical predictors. However, more outcome data are needed to conduct a subgroup analysis.

A high degree of heterogeneity was observed in the reported outcomes. One contributing factor might be that studies had different definitions for significant preoperative ETD scores and cut-off values for clinical improvement. Higgins et al. defined clinically significant ETD as a mean ETDQ-7 item score of at least 2.1. However, this value equated to a total item score cut point of 14.5, which was adopted by the rest of the studies, except for Bowles et al., who used a preoperative score of 13.5 to indicate the presence of ETD [9,23]. The minimal clinically important difference (MCID), the extent of symptom improvement that reached clinical significance, was defined as a reduction in ETDQ-7 score by ≥3.5 postoperatively according to Chang et al. and Chen et al. [18,20], and >3.7 as reported by Hsieh et al. [22]. Higgins et al. assigned this cut-off value at 0.5, whereas the other studies did not provide figures that constituted significant ETD improvement based on the MCID [23]. Second, the etiology of ETD was likely multifactorial, with potential contributors represented by gastrointestinal reflux, allergy, temporomandibular joint disorder, obstructive lesions, or pressure dysregulation, apart from upper respiratory inflammation [30,31]. Since the reported ETD among CRS patients could be attributable to other pathologies, a detailed preoperative evaluation of other predisposing factors to ETD is warranted before study inclusion.

Clinical predictors of ETD improvement following ESS in CRS patients were discussed in previous studies [19,20,23]. A greater preoperative disease burden, delineated by higher ETDQ-7 or SNOT-22 score, revealed a consistent significance in affecting ETD outcome. Higgins et al. indicated that a high preoperative ETDQ-7 score, defined by a mean item score ≥ 4, was negatively associated with ETDQ-7 normalization [23]. A strong link was also found in Chen et al., where a preoperative SNOT-22 score ≥ 40 was correlated to failure of ETDQ-7 score normalization after ESS [20]. In addition, Chen et al. showed that compared with patients who underwent primary surgery, revision surgery was an independent clinical factor in persistent ETD symptoms [19]. Higher inflammatory burden in the sinonasal and middle ear cavity for an extended period thus explained the recalcitrant ETD despite surgical treatments.

Furthermore, Higgins et al. retrospectively explored the variables that were attributed to ETDQ-7 normalization in its study group, and nasal polyposis was one of the preoperative factors that showed a statistical significance to ETDQ-7 normalization [23]. No significant link in regression analysis, however, was found in either Chang et al., Chen et al., or Hsieh et al. [18,19,22]. The inconsistency of statistical significance implied that no definitive conclusions could be drawn regarding the effect of ESS on ETD based on CRS endotypes. Thus, further research is needed to determine whether different inflammatory endotypes contribute to ETD improvement following ESS.

While the surgical efficacy for comorbid ETD might be independent of polyp status, biologic treatment may result in greater improvements in ETDQ-7 scores for patients with CRS with nasal polyps (CRSwNP), which is predominantly characterized by type II inflammation. In particular, one retrospective cohort study reported that Dupilumab, a monoclonal antibody inhibiting interleukin-4 (IL-4) and interleukin-13 (IL-13) pathways, alleviated ETD at a similar magnitude as ESS in Th2-driven CRS [32]. Compared to other biologics, previous studies have proposed Dupilumab as an effective treatment for eosinophilic otitis media (EOM) by relieving Eustachian tube obstruction, with cases of the Eustachian tube opening after Dupilumab having been reported [33]. The pathophysiology behind how Dupilumab resolved CRSwNP and EOM may involve periostin expression in the granulation tissue of nasal polyps and the middle ear [34]. Triggered by type 2 cytokines such as IL-4 and IL-13, periostin is found in thickened mucosa and is thought to prolong inflammation [34,35]. Therefore, by reducing IL-4 and/or IL-13 levels, Dupilumab may inhibit periostin production, thus suppressing inflammation, and consequently improving Eustachian tube obstruction. Since biologics are currently administered as adjunctive therapy for uncontrolled CRSwNP, they are likely to show therapeutic potential in patients with persistent ETD following sinus surgery within the CRSwNP cohort.

There are several limitations to the study. First, although the ETDQ-7 score has been utilized as a reliable clinical instrument for E-tube dysfunction, it is nonetheless reported based on individuals’ subjective symptoms. The result was not supported by comparing with other outcome measure tools, including the Sino-Nasal Outcome Test (SNOT-22), tympanometry, or tubomanometry. Despite not including SNOT-22 as the study measurement, prior studies have consistently shown a strong correlation between ETDQ-7 and SNOT-22 ear subdomains for ETD evaluation [18,21]. A consensus statement released by an European multi-institutional panel of experts has recommended that the diagnosis of Eustachian tube dysfunction be based on both subjective symptoms and objective findings from tympanometry or otoscopy [1]. Although tympanometry demonstrates high sensitivity and specificity for assessing the functional integrity of the middle ear, its effectiveness in diagnosing ETD is limited [36]. Approximately 60% of patients with ETD exhibit normal tympanometry results, highlighting its limitation in detecting mild ETD cases [22]. So far, no universally accepted set of diagnostic means has been validated as a gold standard for measuring ET function. Thus, patient’s subjective reports of improvement in ET function hold greater value for physicians in assessing clinical response. In addition, ETDQ-7 has also been widely implemented for recognizing ETD due to its well-established sensitivity and specificity, as well as its ease of administration [9].

Second, few studies have investigated independent predictors of ESS outcomes on CRS-related ETD, so only parallel comparisons could be drawn from the literature. Lastly, the forest plot was pooled from single-arm observational cohorts, with no control group to compare the outcome of those who did not receive surgery. This could potentially bias the impact of ESS on relieving ETD. Therefore, future randomized controlled trials are encouraged to gain a more accurate understanding of the role of ESS in treating CRS with ETD.

## 5. Conclusions

CRS patients had a high prevalence of concomitant ETD, of which symptoms were significantly improved following ESS at the 3-month postoperative interval. Based on selected studies, greater disease burden, represented by higher preoperative scores or those receiving revision surgery, was shown to be negatively associated with ETD improvement. Larger prospective case studies are encouraged to analyze the change in ETD, stratified by CRS subgroup for statistical purposes. Moreover, future research should incorporate control groups, allowing for a comparison between surgical and conservative treatments to better reflect the actual efficacy of ESS.

## Data Availability

The original contributions presented in the study are included in the article/Supplementary Materials; further inquiries can be directed to the corresponding author.

## Supplementary Materials

The following supporting information can be downloaded at https://www.mdpi.com/article/10.3390/biomedicines12112484/s1, Table S1: PRISMA Checklist; Table S2: Keywords and search results in different databases; Figure S1: The funnel plot of the included studies evaluating the effect of endoscopic sinus surgery on improving ETD symptoms among CRS patients.
